# ntEdit+Sealer: Efficient Targeted Error Resolution and Automated Finishing of Long‐Read Genome Assemblies

**DOI:** 10.1002/cpz1.442

**Published:** 2022-05-14

**Authors:** Janet X. Li, Lauren Coombe, Johnathan Wong, Inanç Birol, René L. Warren

**Affiliations:** ^1^ Canada's Michael Smith Genome Sciences Center Vancouver BC Canada; ^2^ Bioinformatics Graduate Program University of British Columbia Vancouver BC Canada; ^3^ Department of Medical Genetics University of British Columbia Vancouver BC Canada

**Keywords:** assembly finishing, Bloom filter, hybrid assembly polishing, *k*‐mer, long‐read genome assembly

## Abstract

High‐quality genome assemblies are crucial to many biological studies, and utilizing long sequencing reads can help achieve higher assembly contiguity. While long reads can resolve complex and repetitive regions of a genome, their relatively high associated error rates are still a major limitation. Long reads generally produce draft genome assemblies with lower base quality, which must be corrected with a genome polishing step. Hybrid genome polishing solutions can greatly improve the quality of long‐read genome assemblies by utilizing more accurate short reads to validate bases and correct errors. Currently available hybrid polishing methods rely on read alignments, and are therefore memory‐intensive and do not scale well to large genomes. Here we describe ntEdit+Sealer, an alignment‐free, *k*‐mer‐based genome finishing protocol that employs memory‐efficient Bloom filters. The protocol includes ntEdit for correcting base errors and small indels, and for marking potentially problematic regions, then Sealer for filling both assembly gaps and problematic regions flagged by ntEdit. ntEdit+Sealer produces highly accurate, error‐corrected genome assemblies, and is available as a Makefile pipeline from https://github.com/bcgsc/ntedit_sealer_protocol. © 2022 The Authors. Current Protocols published by Wiley Periodicals LLC.

**Basic Protocol**: Automated long‐read genome finishing with short reads

**Support Protocol**: Selecting optimal values for *k‐*mer lengths (*k*) and Bloom filter size (*b*)

## INTRODUCTION

High‐quality genomes have enabled many recent advances in the broader life sciences. Genome assemblies provide a wealth of information for clinical applications, comparative genomics, population studies, and other research areas. Long‐read or third‐generation sequencing technologies from Oxford Nanopore Technologies Ltd. (ONT, Oxford, UK) and Pacific Biosciences of California, Inc. (PacBio) have drastically improved in cost and throughput over the past several years, and these improvements have allowed these technologies to be adopted in a broad range of applications (van Dijk, Jaszczyszyn, Naquin, & Thermes, 2018) and genomics studies (Logsdon, Vollger, & Eichler, 2020). The major advantage of long reads over short Illumina reads, the current gold standard, is their length. Long sequencing reads can span several thousands to millions of nucleotides, while Illumina reads are usually 150‐250 base pairs (bp) long. Long reads provide valuable long‐range genomic information that greatly benefits *de novo* genome assembly projects, resolving repetitive regions to achieve high contiguity (Amarasinghe et al., 2020). However, the appreciable error rate of long reads relative to short reads remains a major limitation to this day, with a mean accuracy of 87%‐99% depending on sequencing platform, chemistry, and base‐calling method (Logsdon et al., 2020).

High base accuracy in genome assemblies is crucial for annotating functional elements and calling variants, for example. Therefore, long‐read genome assemblies often undergo a genome polishing step to improve their base quality. Several genome polishing methods employ a hybrid approach, using more accurate short reads to correct long‐read genome assemblies. Some notable examples of hybrid genome polishing tools include Pilon (Walker et al., 2014) and Racon (Vaser, Sović, Nagarajan, & Šikić, 2017). Both tools rely on read alignments for identifying and correcting erroneous regions. While read alignments are information‐rich, they are also highly memory intensive and therefore not practical for organisms with large genomes. There is a need for scalable and automated tools to polish and finish long‐read genome assemblies.

**Figure 1:**
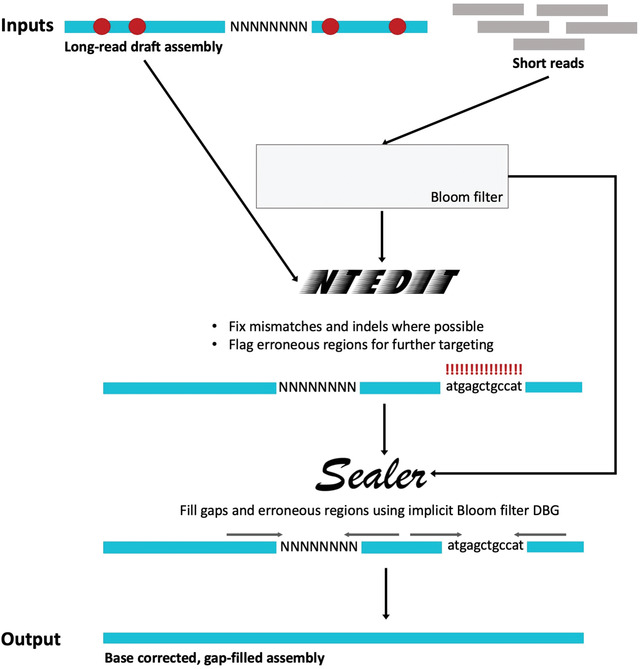
ntEdit+Sealer genome finishing pipeline. ntHits extracts *k*‐mers from short reads and creates a Bloom filter. ntEdit queries assembly *k*‐mers in the short‐read Bloom filter, making base corrections where possible and flagging problematic stretches of sequences. ABySS‐Bloom extracts *k*‐mers and creates another Bloom filter, which is used by Sealer as an implicit de Bruijn graph to fill assembly gaps and problematic regions flagged by ntEdit.

Here we describe a scalable and alignment‐free protocol for correcting base errors and resolving problematic regions in long‐read genome assemblies using short reads. This protocol relies on Bloom filters, a probabilistic data structure that stores and tests for elements in a set in constant time (Bloom, 1970). The protocol includes the assembly correction tools ntEdit (Warren et al., 2019) and Sealer (Paulino et al., 2015), and is called ntEdit+Sealer. Additionally, ntHits (Mohamadi, Chu, Coombe, Warren, & Birol, 2020) and ABySS‐Bloom (Jackman et al., 2017) are required for creating Bloom filters from short read *k*‐mers for the respective tools. A general overview of the ntEdit+Sealer genome finishing protocol is presented in Figure 1. We will illustrate this protocol with an *Escherichia coli* strain NDM5 dataset obtained from the NCBI Sequencing Read Archive (SRA; Accession: SAMN21398207). We provide instructions for installing the required software, both from package managers and manually, in the Strategic Planning section. Additionally, we provide a Support Protocol with guidelines for selecting optimal parameter values for the protocol.

The output of ntEdit+Sealer is a polished long‐read genome assembly with sequence gaps closed. The ntEdit+Sealer protocol provides an accessible solution for correcting errors and producing high quality genome assemblies from long‐ and short‐read sequencing data. This will allow users to take full advantage of the benefits of both technologies. We expect this genome assembly finishing protocol to be invaluable for hybrid sequencing efforts in pursuit of producing contiguous and accurate genome assemblies.

## STRATEGIC PLANNING

### Necessary Hardware

ntEdit+Sealer is a command‐line‐based protocol that requires a 64‐bit Linux or Mac operating system and a sufficient amount of RAM and disk space for generating and storing Bloom filters. Requirements will depend on the genome size of the specific organism under study. The peak memory usage is approximately equal to the size of the Bloom filter generated by ABySS‐Bloom. Guidelines for determining this Bloom filter size are described in the Support Protocol.

### Software Installation

The ntEdit+Sealer genome finishing protocol requires three software packages and their dependencies:

- ntHits v0.0.1+ (https://github.com/bcgsc/nthits)
- ntEdit v1.3.5+ (https://github.com/bcgsc/ntEdit)
- ABySS v2.3.2+; contains Sealer and ABySS‐Bloom (https://github.com/bcgsc/abyss)

All tools can be installed using the Conda or Homebrew package managers, as well as by manually cloning and compiling the source code from Github. The protocol is implemented as a Makefile (GNU Make) pipeline available on Github at https://github.com/bcgsc/ntedit_sealer_protocol.

#### Option A: Installation using the Conda package manager

Installation via the Conda package manager (Miniconda) is recommended. Conda allows easy installation of tools and their dependencies into standalone environments. Miniconda is a minimal, lightweight installation for Conda and can be obtained freely from https://docs.conda.io/en/latest/miniconda.html. Once Miniconda is installed, create a new environment for the protocol with the following commands:

conda create ‐n ntedit_sealer python=3.7
conda activate ntedit_sealer

ntHits, ntEdit and ABySS can then be installed from the bioconda channel:

conda install ‐c bioconda "nthits>=0.0.1" "ntedit>=1.3.5" "abyss>=2.3.2"

This command will install all three tools into the ntedit_sealer environment. This environment must be activated prior to running the protocol with the command conda activate ntedit_sealer.

#### Option B: Installation using the Homebrew package manager

The three packages are also available on Homebrew. The Homebrew package manager can be obtained freely from https://brew.sh. Once Homebrew is installed and configured, the required tools can be installed with the following commands:

brew tap brewsci/bio
brew install nthits ntedit abyss

#### Option C: Manual installation from Github

Since all tools are written in C++, several dependencies are required for compilation:

- Autoconf (https://www.gnu.org/software/autoconf)
- Automake (https://www.gnu.org/software/automake)
- A C++ compiler that supports OpenMP, such as GCC 4.2 or greater (https://gcc.gnu.org)

ABySS requires the following additional libraries:

- Boost (https://www.boost.org)
- OpenMPI (https://www.open‐mpi.org)
- Sparsehash (https://github.com/sparsehash/sparsehash)

##### ntHits

git clone https://github.com/bcgsc/ntHits.git
cd ntHits
./autogen.sh
./configure ‐‐prefix=/path/to/ntHits
make
make install

##### ntEdit

git clone https://github.com/bcgsc/ntEdit.git
cd ntEdit
make ntedit

##### ABySS

git clone https://github.com/bcgsc/abyss.git
cd abyss
./autogen.sh
mkdir build
cd build
../configure ‐‐prefix=/path/to/abyss
make
make install

For ntHits and ABySS, the ‐‐prefix parameter of the configure script defines the path at which the tool will be installed. With sudo privileges, when this parameter is excluded the tool will install into /usr/local.

#### Final steps after all dependencies are installed (i.e., after Option A, B or C)

The ntEdit+Sealer repository must also be downloaded after ensuring that the dependencies have been installed according to options A, B, or C, listed in the section above. The ntEdit+Sealer protocol is implemented as a Makefile pipeline, which is a wrapper for the ntHits, ntEdit, ABySS‐Bloom, and Sealer commands. The repository includes additional scripts required for integrating the tools and processing input files. The scripts can be cloned from Github and do not require any compilation:

git clone https://github.com/bcgsc/ntedit_sealer_protocol.git

To run the tools outside of their directories, their executables must be added to your PATH environment variable:

PATH=/path/to/ntedit_sealer_protocol:/path/to/ntHits/bin:path/to/ntEdit:/path/to/abyss/bin:$PATH

More detailed instructions for installing the packages can be found in their respective Github repositories.

## AUTOMATED LONG‐READ GENOME FINISHING WITH SHORT READS

This Basic Protocol describes how to run ntEdit+Sealer with the protocol Makefile to polish and correct errors in a long‐read genome assembly using short reads. The protocol involves populating several Bloom filters with short‐read *k*‐mers for ntEdit and Sealer using several *k‐*mer sizes. After the Bloom filters are created, ntEdit is run iteratively with long to short *k* values to correct base errors and flag unfixable and problematic regions in the assembly by soft‐masking them. Unmasked sequences that are shorter than the lowest *k* value and flanked by soft‐masked regions are further soft‐masked after the ntEdit runs. Finally, Sealer is run with the same decreasing *k* values to fill in the erroneous soft‐masked regions and existing assembly gaps by traversing an implicit Bloom filter de Bruijn graph.

The pipeline is invoked with a single Makefile command. We illustrate the steps with an *E. coli* strain NDM5 dataset consisting of a long‐read Shasta (Shafin et al., 2020) assembly generated from ONT MinION reads and paired‐end Illumina MiSeq reads for assembly finishing. Additionally, we demonstrate how to analyze the draft and finished assemblies with QUAST (Gurevich, Saveliev, Vyahhi, & Tesler, 2013).

### Necessary Resources

#### Hardware

A server or machine running a 64‐bit Linux or Mac operating system with a sufficient amount of disk space and RAM (see Support Protocol for more details).

### Software

The following packages and their dependencies must be installed and be referenced to in your PATH environment variable:

- sra‐tools v2.9.1+ (https://github.com/ncbi/sra‐tools)
- ntHits v0.0.1+ (https://github.com/bcgsc/nthits)
- ntEdit v1.3.5+ (https://github.com/bcgsc/ntEdit)
- ABySS v2.3.2+ (https://github.com/bcgsc/abyss)
- ntEdit+Sealer protocol v1.0.0+ (https://github.com/bcgsc/ntedit_sealer_protocol)
- QUAST v5.0.0+ (https://github.com/ablab/quast)

### Files

Short sequencing reads (paired‐ or single‐end) can be provided in compressed or uncompressed FASTQ format. Paired‐end reads do not need to be interleaved. The long‐read draft genome assembly can be provided as either a multi‐ or single‐line FASTA file.

### Sample data

The example *E. coli* strain NDM5 draft genome assembly is included in the ntEdit+Sealer Protocol Github Repository under the “demo” subdirectory (https://github.com/bcgsc/ntedit_sealer_protocol/blob/main/demo/ecoli_shasta.fa). The corresponding Illumina short reads will be used as demonstration for polishing and can be obtained from the NCBI Sequencing Read Archive (Accession: SRX12150405). Additionally, we will use the *E. coli* strain K‐12 substr. MG1655 reference genome assembly (Accession: GCF_000005845.2) to assess the assemblies with QUAST.

### Protocol steps

1 Install ntHits, ntEdit, ABySS, and the ntEdit+Sealer repository as outlined in the Strategic Planning section and add all binaries to your PATH environment variable.
2 Install protocol‐specific dependencies fasterq‐dump (part of sra‐tools) and QUAST via Conda or manually. Ensure sra‐tools v2.9.1 or newer is installed in order to obtain fasterq‐dump, a more performant, multi‐threaded version of fastq‐dump. If the correct version cannot be installed, fastq‐dump can be used as a replacement (see step 3 below for details). If installing the tools manually, the executables must be added to your PATH environment variable.

### Option A: Installation using the Conda package manager

If Option A of the Strategic Planning section was used to install ntHits, ntEdit, and ABySS, dependencies may be installed into the same environment. Otherwise, a new Conda environment should be created. The Conda environment must be activated (using conda activate <env name>) prior to installing the tools.

conda install ‐c bioconda "sra‐tools>=2.9.1" "quast>=5.0.0"

### Option B: Manual installation

***sra‐tools***
Identify the correct version of the SRA Toolkit for your operating system from https://github.com/ncbi/sra‐tools/wiki/01.‐Downloading‐SRA‐Toolkit, and replace the URL if necessary.

wget https://ftp‐trace.ncbi.nlm.nih.gov/sra/sdk/3.0.0/sratoolkit.3.0.0‐centos_linux64.tar.gz
tar ‐xzf sratoolkit.3.0.0‐centos_linux64.tar.gz
export PATH=/path/to/sratoolkit.3.0.0‐centos_linux64/bin:$PATH

***QUAST***

wget https://github.com/ablab/quast/releases/download/quast_5.0.2/quast‐5.0.2.tar.gz
tar ‐xzf quast‐5.0.2.tar.gz
export PATH=/path/to/quast:$PATH

3 Create a new directory for running the ntEdit+Sealer protocol. Enter the new directory, soft‐link the draft genome assembly from the ntEdit+Sealer repository and download the reference genome assembly and short reads.
mkdir ecoli_demo
cd ecoli_demo
ln ‐s /path/to/ntedit_sealer_protocol/demo/ecoli_shasta.fa
wget https://ftp.ncbi.nlm.nih.gov/genomes/all/GCF/000/005/845/GCF_000005845.2_ASM584v2/GCF_000005845.2_ASM584v2_genomic.fna.gz
fasterq‐dump SRR15859208
    The fasterq‐dump command will download the short reads into two separate FASTQ files. These files are named according the SRA run accession number (i.e., SRR15859208_1.fastq and SRR15859208_2.fastq for the forward and reverse reads, respectively). If, for some reason, the correct version of sra‐tools and fasterq‐dump cannot be installed, fastq‐dump may be used instead with the following command: fastq‐dump SRR15859208 ‐‐split‐3 ‐‐skip‐technical.
4 Run the ntEdit+Sealer Makefile with the “finish” command to polish and fill the draft assembly ecoli_shasta.fa with the two reads files SRR15859208_1.fastq and SRR15859208_2.fastq. Specify *k*‐mer lengths of 80, 65 and 50 with the k parameter, and a Bloom filter size of 200 MB with the b parameter. The protocol should take approximately 5 min to complete and requires under 550 MB of RAM, so can easily be run on a modern laptop or desktop computer.
ntedit‐sealer finish seqs=ecoli_shasta.fa \
reads="SRR15859208_1.fastq SRR15859208_2.fastq" \
k="80 65 50" b=200M
    Ensure that quotation marks are used to enclose lists of parameter values (i.e., read files and k‐mer lengths), and that individual items in lists are space‐separated. k‐mer lengths must be passed in decreasing order. The command will run (1) ntHits and ABySS‐bloom to populate Bloom filters from the short reads, (2) a Bash script to call ntEdit iteratively with decreasing k, (3) a Python script to consolidate (soft‐mask) sequences shorter than the lowest k that are flanked by soft‐masked regions, and finally (4) Sealer.
5 Ensure that the ntEdit+Sealer run completes successfully. Successful completion will result in the Makefile reporting “ntEdit and Sealer polishing steps complete! Polished assembly can be found in: ecoli_shasta.ntedit_edited.prepd.sealer_scaffold.fa”.
6 Run QUAST to analyze the draft and ntEdit+Sealer‐finished genome assemblies.
quast ‐‐fast ‐r GCF_000005845.2_ASM584v2_genomic.fna.gz \
‐o ecoli_quast ecoli_shasta.fa \
ecoli_shasta.ntedit_edited.prepd.sealer_scaffold.fa
    All QUAST output files will be printed to the ecoli_quast directory, specified by the ‐o parameter. A summary report will be printed in tab‐separated format to a file named report.tsv, where each column describes one of the input assemblies. The “# mismatches per 100 kbp” metric in this summary describes the base accuracy of the draft and finished assemblies compared to the reference. The “# indels per 100 kbp” metric describes the average proportion of insertions or deletions of either assembly compared to the reference. The expected values for these metrics are shown in Table 1. If QUAST was installed manually, the executable will be quast.py.

**Table 1:** Number of Mismatches and Indels per 100 kbp for *E. coli* Assembly Before and After Finishing with ntEdit+Sealer^*a*^

	Draft assembly	After ntEdit+Sealer
# mismatches per 100 kbp	371.33	345.86
# indels per 100 kbp	122.38	7.22

^*a*^ Running ntEdit+Sealer assembly finishing protocol decreases the proportion of mismatched bases and the proportion of indels in the *E. coli* genome assembly.

## SELECTING OPTIMAL VALUES for *k‐*mer LENGTHS (*k*) AND BLOOM FILTER SIZE (*b*)

Both ntEdit and Sealer employ a *k*‐sweep approach, iterating from long to short *k*‐mer lengths. This approach is beneficial because different *k*‐mer lengths can provide resolution at different scales. Larger *k*‐mers can disambiguate repeats as they span longer regions, while shorter *k*‐mers are useful when the local read coverage is low and for assemblies with lower base quality. The same sequence of *k* values is used for both tools. *k*=40 is the practical lower limit for Sealer, as shorter *k* values cause its runtime to increase sharply. We find that *k*=80 generally performs well for a variety of datasets and suggest decreasing in intervals of 10‐15. Generally speaking, there is no strict upper limit for *k* (apart from the read length), so a wide range of *k*‐mer lengths can be used to achieve the best polishing results. Time and memory restrictions will be the limiting factors in these cases.

ntHits will automatically select the optimal Bloom filter size for ntEdit by calculating the *k*‐mer distribution for the input reads, but ABySS‐bloom requires the desired Bloom filter size to be specified. This parameter is controlled by the b parameter when invoking the protocol Makefile.

The optimal size of a Bloom filter depends on several factors, namely the desired false positive rate (FPR), number of hash functions used for insertion, and number of elements that will be inserted. For large Bloom filters, the FPR can be approximated (Equation 1):

(1) $$f={\left(1-e^{\frac{-hn}{m}}\right)}^{h}$$

where *f* is the FPR, *m* is the size of the filter in bits, *h* is the number of hash functions, and *n* is the number of elements (Broder & Mitzenmacher, 2004). By default, ABySS‐Bloom uses one hash function for insertion. Using this relationship and asserting *h*=1, we can approximate the optimal *m* for a given dataset and desired FPR (Equation 2):

(2) $$m=Ceil\left(\frac{-n}{\ln\left(1-f\right)}\right)$$

The following Bloom filter sizes (RAM) generally perform well for common model organisms:

- *Homo sapiens* (3 Gbp genome): 100 GB
- *Caenorhabditis elegans* (100 Mbp genome): 2.5 GB
- *Escherichia coli* (5 Mbp genome): 200 MB

The optimal b value for other genome sizes can be interpolated from these guidelines, or can be estimated using ntCard (Mohamadi, Khan, & Birol, 2017). ntCard is a streaming algorithm for estimating *k*‐mer frequencies in genomic datasets and can be used to determine the number of unique *k*‐mers in a set of short reads. ABySS‐Bloom creates a 2‐level cascading Bloom filter (Salikhov, Sacomoto, & Kucherov, 2014) from short‐read *k*‐mers; this means that only *k*‐mers appearing two or more times are accounted for. Therefore, only *k*‐mers with multiplicity of 2 or more should be considered when estimating optimal Bloom filter size.

The following steps in this Support Protocol describe how ntCard should be used to calculate the optimal Bloom filter size for a dataset, ensuring a false positive rate (FPR) of ∼0.005. The same *E. coli* short reads from the Basic Protocol will be used to demonstrate this protocol.

### Necessary Resources

#### Hardware

A server or machine running a 64‐bit Linux or Mac operating system capable of running ntCard.

### Software

ntCard v1.2.2+ (https://github.com/bcgsc/ntCard)
*ntCard is available on the Conda and Homebrew package managers and can also be cloned and compiled from Github*.

### Files

The short sequencing reads that will be used as input to ntEdit+Sealer will be analyzed here. The reads can be provided in compressed or uncompressed FASTQ format and paired‐end reads do not need to be interleaved.

1 Install ntCard.

### Option A: Installation using the Conda package manager

conda create ‐n ntcard
conda activate ntcard
conda install ‐c bioconda "ntcard>=1.2.2"

### Option B: Installation using the Homebrew package manager

brew install brewsci/bio/ntcard

### Option C: Manual installation from Github

git clone https://github.com/bcgsc/ntCard.git
./autogen.sh
./configure ‐‐prefix=/path/to/ntCard
make
make install
  The ‐‐prefix parameter for the configure script installs ntCard to the provided path. This parameter can be excluded if you have sudo privileges and wish to install the tool into /usr/local. If using Conda, activate the environment that ntCard was installed to with the command conda activate environment_name. If manually installing ntCard to a specific directory, ensure that the path supplied to the ‐‐prefix parameter is on your PATH.

2 Run ntCard on the read files, providing all *k* values you are planning on using for ntEdit+Sealer. We will use *k*=80, *k*=65, and *k*=50.
ntcard ‐k80,65,50 ‐p freq \
SRR15859208_1.fastq SRR15859208_2.fastq
    This command will generate a k‐mer distribution histogram for each k provided. Each histogram will be printed to a two‐column, tab‐separated file with the prefix freq, for example, freq_k80.hist, where the first column represents an F_k_ metric or multiplicity and the second column contains the number of corresponding k‐mers.
3 Inspect the *k*‐mer frequency histograms. Only the first three lines are necessary for our purposes.
head ‐n 3 freq_k*.hist
    The first two lines of each histogram contain F_k_ metrics, which describe statistics for the input dataset. F0 is the number of distinct k‐mers in the reads, and F1 denotes the total number of k‐mers in the dataset. The third line contains the number of k‐mers appearing once in the reads. See Table 2 for the expected values of these metrics.

**Table 2:** F1, F0 and Number of Multiplicity 1 *k*‐mers for *E. coli* Illumina Reads^*a*^

*k*	F1	F0	1
50	322,869,315	47,853,592	41,155,307
65	300,812,763	50,900,032	44,179,082
80	278,838,102	52,274,328	45,577,189

^*a*^ Table values correspond to the first three rows of ntCard output histograms for 50‐mer, 65‐mer, and 80‐mer cardinality estimation of *E. coli* strain NDM5 Illumina reads.

4 Estimate the number of *k*‐mers that appear two or more times in the reads. Subtract the number of *k*‐mers that appear only once from F0 with the following one‐liner:
for k in 80 65 50; do distinct=$(grep “F0” freq_k$k.hist | awk '{print $2}'); once=$(grep “˄1\b” freq_k$k.hist | awk '{print $2}'); echo k=$k: n=$((${distinct} ‐ ${once})); done
    This command will calculate and print the number of k‐mers appearing two or more times for each k. See Table 3 for the expected values for each k.

**Table 3:** Number of *k*‐mers Appearing at Least Twice in *E. coli* Illumina Reads^*a*^

*k*	n
50	6,697,139
65	6,720,950
80	6,698,285

^a^ Number of *k*‐mers with multiplicity of 2 or more calculated from ntCard output. For each *k*, n is equal to the number of unique *k*‐mers in the dataset (F0) minus the number of *k*‐mers with multiplicity of one.

5 Calculate the optimal Bloom filter size from the *k*‐mer distributions using the largest *n* calculated in step 4 (*k*=65). This will maximize the number of unique *k*‐mers considered, and therefore produce the lowest FPR for the range of *k*, a method analogous to that employed by Kmergenie (Chikhi & Medvedev, 2013). Using the relationship between FPR and Bloom filter density, the optimal value of *m* given an FPR (*f*) of 0.005 is (Equation 3):
(3) $$m=Ceil\left(\frac{-n}{\ln\left(1-f\right)}\right)=Ceil\left(\frac{-6720950}{\ln\left(0.995\right)}\right)=1340826718\;\mathrm{bits}$$

This equates to approximately 168 MB. We rounded this estimate up to 200 MB for the example in the Basic Protocol.

## GUIDELINES FOR UNDERSTANDING RESULTS

The final output of ntEdit+Sealer is a base‐corrected assembly with assembly gaps closed. The file will have the suffix .ntedit_edited.prepd.sealer_scaffold.fa. The Makefile will exit with a non‐zero code if an error occurs during any of the intermediate steps. Otherwise, the pipeline will log a success message to inform the user of its completion.

ntEdit keeps track of the base corrections made to the draft assembly, and these changes are printed for each iterative run to a tab‐separated file with the suffix _changes.tsv. Each iterative run will also produce an intermediate edited FASTA file. Sealer will print information about how many gaps are closed during each iterative *k* to a single file with the suffix .sealer_log.txt.

Genome assembly assessment tools such as BUSCO (Simão, Waterhouse, Ioannidis, Kriventseva, & Zdobnov, 2015) and QUAST (Gurevich et al., 2013) can be used to assess assembly correctness before and after polishing. BUSCO is a tool that quantifies the completeness of genome assemblies, transcriptomes, or gene sets with sets of evolutionarily conserved, single‐copy genes. These gene sets are referred to as Benchmarking Universal Single‐Copy Orthologs (BUSCOs) and are available for many clades across the tree of life. The presence, absence, or duplication of BUSCO members within a genome assembly provides a metric for how complete the assembly is in genic space. Polishing with ntEdit+Sealer improves the base quality and therefore should increase the percentage of complete BUSCO genes in your draft assembly. Since BUSCO only searches for conserved genes within a genome assembly, it is a favorable solution when performing *de novo* assembly for an organism without a reference genome. QUAST is a genome assembly quality‐assessment tool that produces a wide range of metrics and visualizations and is particularly beneficial when a reference genome is available. Of note, the number of mismatches per 100 kbp and number of indels per 100 kbp describe the accuracy of your genome assembly in relation to the reference. These metrics should decrease after using ntEdit+Sealer to polish and finish your draft genome, assuming the assembled genome is highly similar to the reference genome.

## COMMENTARY

### Background Information

ntEdit was developed as an assembly polishing tool (Warren et al., 2019), and Sealer was developed as a tool for filling gaps in genome assemblies (Paulino et al., 2015). New features (discussed in the Critical Parameters section) have been implemented in both tools that allow them to work harmoniously to resolve assembly errors. The multi‐step approach allows error correction at different levels of resolution. ntEdit resolves assembly errors on a small scale, correcting base errors and insertions and deletions up to 5 nucleotides in length by querying the short‐read Bloom filter for all possible edits. If none of the possible edits have sufficient support, the erroneous region is soft‐masked for further base correction by Sealer. Sealer fills the larger erroneous assembly regions, as well as existing hard‐masked assembly gaps, by traversing an implicit Bloom filter de Bruijn graph to find a path that connects the gap‐flanking sequences.

Both tools employ Bloom filters, making them more memory efficient and quicker than alignment‐based approaches. Bloom filters only consist of an array of bits, thereby requiring less memory during runtime and storage. Additionally, inserting and querying for elements in Bloom filters are both constant time operations, depending only on the number of hash functions used for insertion. These characteristics make Bloom filter–based tools beneficial for storing and querying large sequencing datasets and allow the ntEdit+Sealer protocol to be accessible to groups without access to large amounts of RAM and processing power.

### Critical Parameters

When working with Bloom filters, the Bloom filter size and *k*‐mer length are of critical importance. The Support Protocol provides guidelines for selecting the optimal values for these parameters. ntEdit and Sealer must be configured to be compatible with their respective soft‐masking parameters. The soft‐masking behavior of ntEdit is controlled by setting ‐a 1. Sealer does not recognize lower‐case (soft‐masked) characters as assembly gaps by default; the ‐‐lower flag dictates this behavior. The ntEdit+Sealer Makefile has these behaviors configured automatically.

### Troubleshooting

The Makefile should not finish running (i.e., will exit with a non‐zero status) if an error occurs during any of the intermediate steps. Carefully inspect the logs, output files, and error codes if an error occurs. See Table 4 for possible problems, causes and solutions.

**Table 4:** Sources of and Solutions to Potential Errors^*a*^

Problem	Possible cause	Solution
ntEdit does not make any edits and/or Sealer does not fix any regions	The supplied *k* values may be too low or high	Sweep a larger range of *k* values. For ntEdit, check the _changes.tsv output files to determine which *k* values produce the most edits. For Sealer, check .sealer_log.txt file to determine which *k* values close the most gaps. Adjust the parameter values accordingly.
Sealer step takes too long	The provided *k* value is too low	The realistic lower‐bound for *k* is 40. Run the protocol again with higher *k* values.
Assembly accuracy metrics from BUSCO or QUAST deteriorate	False positive rate of ABySS‐Bloom Bloom filter may be too high.	Increase the Bloom filter size (b parameter) or try larger *k* values
The ntEdit+Sealer Makefile gives the error “No rule to make target…”	Parameter values or files were passed or named incorrectly	Ensure input files are named correctly and that parameters are passed as expected. Run ntedit‐sealer help or check the ntEdit+Sealer Github page for more details.

^a^ Potential problems that a user may encounter when running ntEdit+Sealer protocol and corresponding potential causes and solutions.

If other problems arise related to the ntEdit+Sealer protocol Makefile and scripts, create a Github Issue on the ntEdit+Sealer Github page at https://github.com/bcgsc/ntedit_sealer_protocol/issues. Please address any problems related to specific tools on their respective Github Issues pages.

### Author Contributions

**Janet X. Li**: software, writing original draft; **Lauren Coombe**: software, validation, writing review and editing; **Johnathan Wong**: software, writing review and editing; **Inanç Birol**: supervision, writing review and editing; **René L. Warren**: conceptualization, software, supervision, writing review and editing.

### Conflict of Interest

The authors declare that they have no competing interests.

## Data Availability

The example data used in the Basic Protocol and Support Protocol are openly available. The *E. coli* strain NDM5 Illumina short reads are available from the NCBI SRA at https://trace.ncbi.nlm.nih.gov/Traces/sra/?run=SRR15859208 (Accession: SRR15859208). The draft long‐read assembly was generated from MinION reads which are available from the SRA at https://trace.ncbi.nlm.nih.gov/Traces/sra/?run=SRR15859207 (Accession: SRR15859207). The draft assembly can be found at https://github.com/bcgsc/ntedit_sealer_protocol/blob/main/demo/ecoli_shasta.fa and the *E. coli* reference assembly is available at https://www.ncbi.nlm.nih.gov/assembly/GCF_000005845.2 (Accession: GCF_000005845.2).
